# Digital technology empowers model innovation in chronic disease management in Chinese grassroots communities

**DOI:** 10.3389/fdgth.2025.1726932

**Published:** 2026-01-02

**Authors:** Quansheng Wang, Ziqing Zhang, Guoqing Han

**Affiliations:** Discipline Inspection and Supervision School, Shandong University, Weihai, China

**Keywords:** chronic, digital, diseases, health, information construction, management, model innovation, noncommunicable

## Abstract

China is one of the countries with a heavy burden of chronic diseases in the world. The number of people with chronic diseases and the number of deaths show a continuous, rapid growth trend. In recent years, China has actively promoted the innovation of digital management models for chronic diseases in grassroots communities, improved the allocation of community resources, clarified service specifications, strengthened information construction, and established a supervision and evaluation system. Digital management measures for chronic diseases in grassroots social areas include building a digital health management platform, promoting the popularization and application of intelligent detection equipment, and carrying out mobile medical and telemedicine services. However, it also faces some challenges, such as data security, privacy protection, uneven technology application, and patient acceptability. China should propose targeted measures to address these challenges, further promote the digital management of chronic diseases in grassroots communities, and protect the lives and health of people.

## 1 Introduction

Chronic noncommunicable diseases (NCDs), or long-term chronic diseases, include cardiovascular and cerebrovascular diseases such as hypertension, stroke, and coronary heart disease, as well as diabetes, mental illness, and malignant tumors (1). They are characterized by a long course of disease, complex etiology, and serious social and health damage (2). The World Health Organization noted in 2016 that there are four main types of chronic diseases: cardiovascular diseases, cancer, respiratory diseases, and diabetes (3). China is one of the countries with the heaviest burden of chronic diseases in the world today. Data for 2023 show that there are currently 245 million people with hypertension in China, and the prevalence of hypertension among residents over 18 years of age reaches 27.5%; the number of people with diabetes has reached 140 million; the number of people with abnormal blood lipids exceeds 400 million, with a prevalence rate of 40.4%; and the number of overweight or obese people exceeds 500 million (4). In China, chronic diseases, mainly hypertension and diabetes, show the phenomenon of “three lows and one high”, that is, low awareness rate, treatment rate, and control rate, and high prevalence rate (5).

Traditional treatment of chronic diseases in the community is based primarily on medical personnel from community health service centers who make regular home visits, and patients who visit the center for treatment. In this model, medical personnel have a substantial workload, making it difficult to provide refined treatment to a large number of patients with chronic diseases (6). At the same time, patients' attention to their own diseases and their ability to self-manage vary, and some patients cannot take medication on time or monitor their condition regularly, leading to poor disease control. Furthermore, due to poor information communication, there is a problem of information disconnect when patients are referred from community health service centers to higher-level hospitals, which affects the continuity and effectiveness of treatment (7). In addition, China's chronic disease management has long been dominated by in-hospital management, and there is a lack of systematic out-of-hospital management after patients leave the hospital. And it is difficult to effectively link and track hospital and outpatient disease data (8). Although some communities have carried out electronic chronic disease management archive compilation work, due to the large workload and low data quality, they have not been fully engaged in their duty (9).

The rapid development of digital technology has brought new opportunities for chronic disease management. This study aims to explore how digitalization empowers the innovation of community chronic disease management models, analyze existing practical cases, and propose future development suggestions. Through literature review and case analysis methods, this article systematically sorts out the state of the application and future trends of digitalization in chronic disease management.

## 2 Methodology

This study employs a comprehensive research approach that combines a systematic review of the literature with a case study analysis to investigate the application of digital technology in the treatment of chronic diseases within grassroots communities in China. The methodology is designed to ensure a comprehensive and multifaceted understanding of the topic by triangulating data from policy documents, practical implementations, and academic literature.

The search for policy documents was carried out between January 2013 and December 2024, a period that captures the significant acceleration of China's digital health initiatives. Primary sources included the official portal of the Chinese Central People's Government (https://www.gov.cn), websites of the National Health Commission and other relevant ministries, as well as official government websites of 31 administrative regions at the provincial level. The search strategy used a combination of Chinese keywords, such as “digitalization”, “chronic disease management”, “community”, and “primary healthcare”. This process identified 5,851 relevant documents, which were then screened to focus on those explicitly highlighting digital strategies for chronic disease management at the community level, leading to an analysis of key central and local policies.

For practical cases and academic literature, we searched databases that included China's National Knowledge Infrastructure (CNKI), Wanfang Data, PubMed, and Web of Science. Search terms included (“digital technology” OR “smart device”) AND (chronic disease' OR “hypertension” OR “diabetes”) AND (community management' OR “primary healthcare”) in English and Chinese. The inclusion criteria required that studies or reports clearly describe the application of digital tools for the management of chronic diseases in a community setting within China. The exclusion criteria were studies focused solely on hospital management, not involving digital technology, or outside of the Chinese context. This process allowed for the identification and in-depth analysis of representative cases, such as those in Qingdao, Beijing, and Shenzhen, to illustrate the models discussed.

## 3 Leadership in policy and innovation in digital management models for chronic diseases in the community

Policies play a dual role of “steering wheel” and “booster” in the management of community chronic diseases. They not only clarify the direction and delineate the framework through top-level design, but also promote implementation through resource allocation, mechanism innovation, supervision, and evaluation, and ultimately achieve the goal of “early prevention, early detection, early intervention, and whole-process management” of chronic diseases.

### 3.1 Top-Level design of central policies

Through strategic planning and institutional design, the community is placed at the core of chronic disease prevention and control, providing a “coordinate system” for management work. The central policy is relatively macroeconomic, mainly establishing the top-level design of the digital management model for chronic diseases in grassroots communities in China through strategic guidance and division of responsibilities (see Table 1).

**Table 1:** Statistics on the policies of the central government of China on chronic disease management in digitally enabled communities.

No	Name of the policy	Formulation body	Date of implementation	Main content
1	Report on Promoting the Construction of Old-age Service System, Strengthening and Improving the Care of Disabled Elderly	The State Department	September 10, 2024	China will accelerate the formation of an old-age service system that is compatible with actively responding to the aging of the population and promoting Chinese-style modernization. China will improve digital applications and services for the elderly and provide a safer, more comfortable, and more convenient digital and intelligent living environment for the elderly.
2	Opinions on Further Perfecting the Medical and Health Service System	General Office of the CPC Central Committee	March 23, 2023	Strengthen the ability of diagnosis and treatment of common and frequently occurring diseases, public health, health management, and traditional Chinese medicine services, improve the level of infectious disease screening, prevention, and control, strengthen the health management of major chronic diseases, carry out mental health guidance for residents, and enhance the medical service capacity of township hospitals such as routine surgery at or below the second level.
		General Office of the State Council		
3	Opinions on Further Deepening the Reform and Promoting the Healthy Development of the Rural Medical and Health System	General Office of the CPC Central Committee	February 23, 2023	By 2025, remarkable progress will have been made in the reform and development of the rural medical and health system. The functional layout of rural health and medical institutions is more balanced and reasonable, the infrastructure conditions are improved, the application of intellectualization and digitalization is gradually popularized, the advantages of traditional Chinese medicine are further developed, the ability of disease prevention and treatment and health management is significantly improved, and the ability to deal with major epidemics and public health emergencies in rural areas is continuously enhanced.
		General Office of the State Council		
4	Notice on Issuing the Implementation Plan of Major Projects for the Revitalization and Development of Traditional Chinese Medicine	General Office of the State Council	February 10, 2023	Research and development of digital auxiliary diagnostic equipment of traditional Chinese medicine, and demonstration of the application of traditional Chinese medicine technology and equipment in the prevention and control of chronic diseases.
5	Notice on Printing and Issuing the 14th Five-Year Plan for the Development of Traditional Chinese Medicine	General Office of the State Council	March 3, 2022	China will build a high-quality and efficient service system of traditional Chinese medicine and give full play to its role in the prevention and treatment of common diseases, frequently occurring diseases, and chronic diseases.
6	Notice on Printing and Issuing the 14th Five-Year Plan for the Construction of Urban and Rural Community Service System	General Office of the State Council	December 27, 2021	Efforts should be made to improve the service capacity of grass-roots health care and medical care, and to do a good job in the prevention and control of infectious diseases, chronic diseases, and children's health.
7	Notice on Issuing the National Action Plan for Disability Prevention (2021–2025)	General Office of the State Council	December 14, 2021	China should strengthen the prevention and control of disability caused by chronic diseases and promote the integrated management of medical treatment and prevention of chronic diseases at all levels.
8	Notice on Printing and Issuing the “14th Five-Year” Plan for Universal Medical Security	General Office of the State Council	September 23, 2021	Optimize the coordinated management system of medical security and strengthen the management of chronic diseases.
9	Notice on Issuing the National Fitness Program (2021–2025)	The State Department	July 18, 2021	Digital upgrade and transformation of national fitness facilities.
10	Notice on Issuing the 14th Five-Year Plan for the Protection and Development of Disabled Persons.	The State Department	July 8, 2021	Implement chronic disease prevention and intervention measures, adhere to the principles of traditional service mode and intelligent service innovation, establish an online and offline service system for the disabled, and promote the inclusive application of digital services in disability assistance.
11	Opinions on Strengthening the Modernization of the Grass-roots Governance System and Governance Capacity	Central Committee of the Chinese Communist Party*******The State Department	April 28, 2021	China will promote the construction of comprehensive service facilities in urban and rural communities, relying on them to provide employment, old age care, medical care, childcare, and other services, strengthen care for the disadvantaged groups and special groups, and do a good job in the prevention and control of infectious diseases and chronic diseases.
12	Opinions on Promoting the Healthy Development of Care Services for the Aged	The State Department	December 14, 2020	Innovative development of health consultation, emergency rescue, chronic disease management, life care, goods purchase, and other intelligent health care services.
13	Opinions on Promoting the Inheritance, Innovation, and Development of Traditional Chinese Medicine	The CPC Central Committee and the State Council	October 20, 2019	Promote the inheritance and open and innovative development of traditional Chinese medicine, promote the digital transformation of traditional Chinese medicine techniques, and promote the application of traditional Chinese medicine in chronic disease management.
14	Opinions on Promoting the Development of “Internet + Medical Health”	General Office of the State Council	April 25, 2018	China will improve the “Internet + medical and health” service system, focusing on hypertension and diabetes, and strengthen the management of online services for chronic diseases in the elderly.
15	Outline of the National Innovation-Driven Development Strategy	The CPC Central Committee and the State Council	May 7, 2016	Build digital infrastructure and develop digital medical care.
16	Notice on Issuing the Outline of the outline of the strategic plan for the development of traditional Chinese Medicine (2016–2030)	The State Department	February 22, 2016	Give full play to the prevention and treatment functions of traditional Chinese medicine in common diseases, frequently occurring diseases, and chronic diseases.
17	Report on the Progress of Deepening the Reform of the Medical and Health System	The State Department	December 22, 2015	Focusing on improving the ability of primary medical services and taking the graded diagnosis and treatment of common diseases, frequently occurring diseases, and chronic diseases as breakthroughs, we will gradually guide the sinking of high-quality medical resources.
18	Some Opinions on Promoting the Development of the Health Service Industry	The State Department	September 28, 2013	China will vigorously develop medical services and improve the ability of the community to provide daily care and chronic disease management services for the elderly.

#### 3.1.1 Strategic guidance

Central-level policies [such as the “Healthy China 2030” planning outline and the Medium and Long-Term Plan for the Prevention and Control of Chronic Diseases in China (2017–2025)] clearly propose a “community-based” chronic disease prevention and control strategy, incorporating the community management of key chronic diseases such as hypertension and diabetes into the national health priority development area and establishing the core goals of “reducing incidence and improving control rates.” (10).

#### 3.1.2 Division of responsibilities

Central policies clarify the responsibilities of entities such as community health service centers, disease control centers, general hospitals, and neighborhood committees (such as communities being responsible for daily follow-up visits, hospitals being responsible for difficult and complex diagnoses and treatments, and disease control departments being responsible for data monitoring), avoiding “multiheaded management” or “responsibility vacuum.” For example, the Chinese Basic Public Health Service Project clearly stipulates that communities should undertake the standardized treatment of patients with hypertension and diabetes within their jurisdictions, forming a division of labor system of “primary care at the grassroots level and two-way referral.” (11).

### 3.2 Specific planning of local policies

In addition to central decision-making bodies such as the Central Committee of the Communist Party of China, the State Council, and the National Health Commission that formulate macro policies and strengthen top-level design, China also needs the active cooperation of various localities to introduce specific opinions on the implementation of policies to promote the implementation of policies (12).

**Figure 1:**
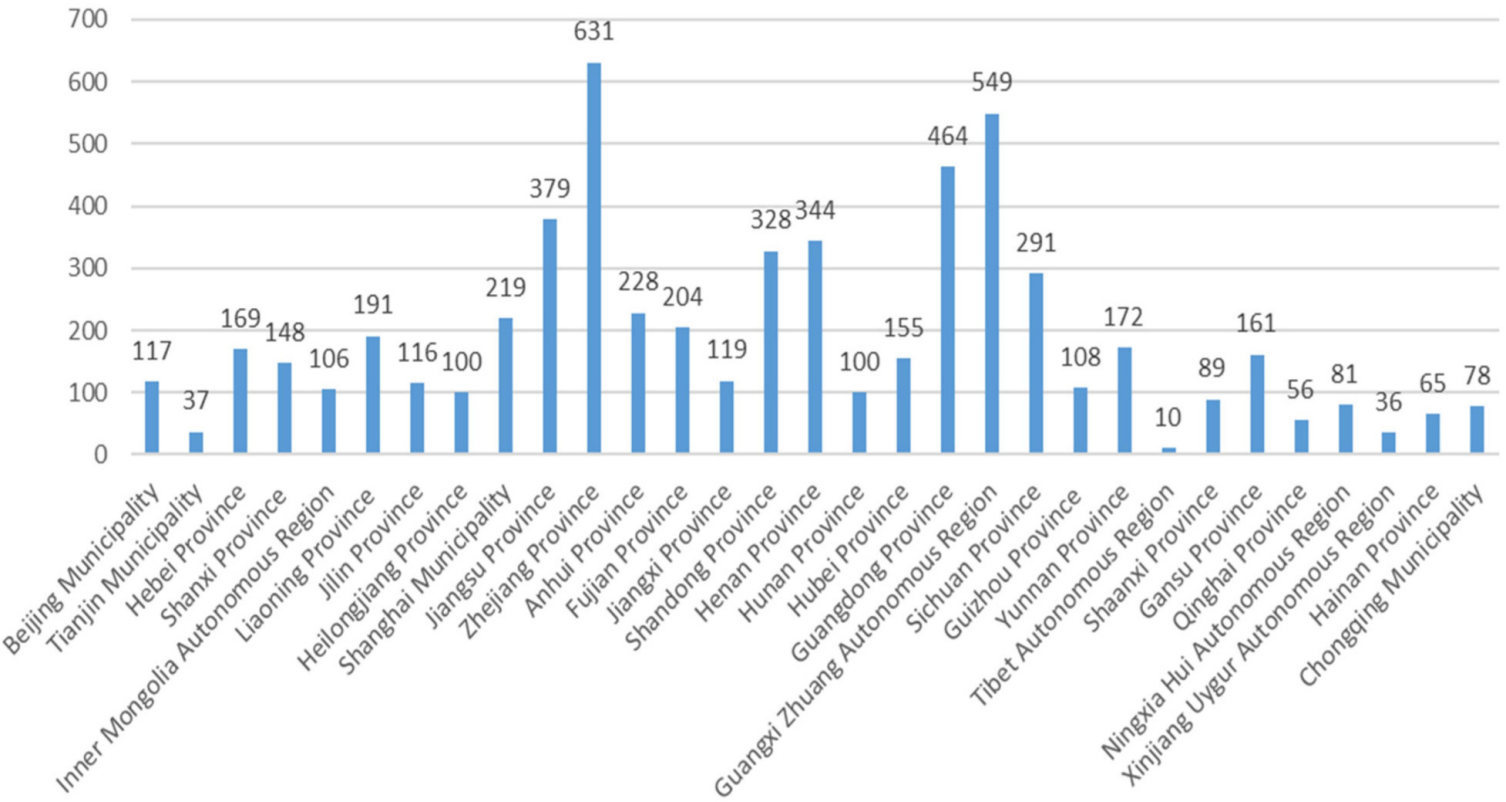
Statistics on the number of policy documents issued by various provinces in China on the use of digital technology to empower the management of community-based chronic diseases.

According to the keywords “digitalization”, “chronic disease management”, and “community” set in the Chinese local government website search, to implement the central policy, local governments have successively issued about 5,851 specific regulations, rules, and normative documents according to the actual conditions of the local area, which concretize the top-level policy design and promote the implementation of the central policy. 3 1 Provinces and cities in mainland China have formulated relevant policy documents (see Figure 1).

## 4 The basis for the application of digital technology in the management of community chronic diseases

The realization of chronic disease management in the digital community depends on the collaborative application of several key technologies. The Internet of Things technology realizes the real-time collection and transmission of patient physiological parameters through wearable devices and home monitoring devices, providing a basic condition for remote monitoring. Big data analysis technology can process massive amounts of health data and identify potential health risks and development trends through machine learning algorithms. The cloud computing platform provides an elastic and scalable infrastructure for data storage and processing, ensuring the security and accessibility of medical data. Mobile health applications enable patients to access their health data anytime, anywhere, receive reminders and content from health education, and improve patient participation and compliance. The integrated application of these technologies provides solid technical support to build a comprehensive and continuous chronic disease management system (13).

Through the top-level design of the central policy and the specific promotion of local policies, China has initially realized the wide application of digital technology in community chronic disease management, and has shown the following results.

### 4.1 Construction of a digital health management platform

In order to improve the current situation of chronic disease management in communities, various regions have explored the construction of digital health management platforms. These platforms integrate resident health records, diagnosis and treatment information, physical examination data, and other multisource information, allowing centralized management of chronic disease patient information (14). For example, the “Intelligent” Health Consortium Chronic Disease Management Platform built by Qingdao West Coast New Area District Hospital is associated with public health systems, HIS systems, blood glucose systems, ECG systems, etc., based on the principle of “three-level coordinated development”, a new chronic disease management model has been established. Patients' blood glucose, blood pressure, and other data measured in medical institutions or at home can be uploaded to the platform in real time. The platform automatically generates red, yellow, and green classification health records and cardiovascular high-risk groups according to the three-level standards of blood glucose management, which greatly improves record creation efficiency and data authenticity and precision (15).

### 4.2 Popularization and application of intelligent monitoring equipment

The emergence of intelligent monitoring equipment, such as intelligent blood pressure monitors, blood glucose meters, and wristbands, has provided convenience for self-monitoring by patients with chronic diseases. These devices can be connected to patients' mobile phones or digital health management platforms through technologies such as Bluetooth and Wi-Fi to realize automatic data upload and real-time sharing (16). For example, the “AI-Powered Precision Management of Hypertension Project” launched by Gulou Community Health Service Center in Miyun District, Beijing, equipped 117 hypertension patients with intelligent blood pressure monitors. The blood pressure monitor can be directly connected to the patient's mobile phone, and the measurement data is uploaded to the mobile phone and the AI management system in real time. Patients and medical personnel can check blood pressure changes at any time, realizing joint monitoring and management by doctors and patients. When the patient's blood pressure is continuously high, the AI system will automatically identify and trigger a grading response mechanism, on the one hand, sending reminder text messages to the patient and initiating a call to remind the patient to pay attention to their health; on the other hand, notifying the medical staff to follow up and provide professional advice, effectively improving the management effect of patients with hypertension (17).

### 4.3 Preliminary development of Mobile medical and telemedicine services

Mobile medical applications (apps) and telemedicine services have also been widely used in the treatment of chronic diseases in communities. Patients can communicate online with family doctor teams through mobile phone applications, consult about their conditions, obtain medication guidance, and make appointments for diagnosis and treatment. Telemedicine uses video communication technology to conduct remote consultations between experts from higher-level hospitals and community medical personnel and patients, allowing patients to enjoy high-quality medical services at their doorsteps (18). For example, in some remote communities, patients can transmit their examination data to experts at higher-level hospitals in real time through telemedicine equipment, and experts can formulate personalized treatment plans for patients based on the data, improving the accessibility and fairness of medical services (19).

## 5 Typical models of digital empowerment for the innovation of community chronic disease management

Various places have explored digital empowerment models for the management of community chronic diseases. Some experiences can be replicated, but creating their own characteristic models is still the direction of efforts in various places. When looking at these innovative models, there are mainly the following three types:

### 5.1 “AI + chronic disease management” model

The application of artificial intelligence technology in the management of community chronic disease provides strong support for precision management. AI systems can analyze a large amount of patient health data, mine the patterns and potential risks behind the data, and achieve accurate prediction and evaluation of the condition of patients with chronic diseases (20). Taking diabetes management as an example, a notable implementation is the “Ruitang” smart management system, piloted in a community health service center in Shanghai's Changning district. This AI system goes beyond simple data aggregation; it integrates continuous glucose monitoring (CGM) data, dietary logs analyzed through image recognition, exercise tracker information, and medication adherence records. Using machine learning algorithms, the system analyzes the multifaceted causes of blood glucose fluctuations for each patient and provides highly personalized recommendations, such as suggesting specific low-glycemic food alternatives available in local supermarkets or adjusting exercise intensity based on real-time glucose trends (21). At the same time, the AI monitors the patient's condition in real time. When it detects abnormal blood glucose fluctuations or a high risk of complications, it issues tiered early warnings. For example, a minor deviation could trigger an automated on-app message suggesting a walk, while a persistent critical level would immediately alert the patient, his family, and the community doctor, prompting a telemedicine consultation within the hour. A study of this system showed that it successfully increased the rate of blood glucose control among diabetic patients by 20% and reduced the incidence of acute complications by 35% through proactive data-driven interventions (22).

### 5.2 Internet + family doctor contracted service model

The deep integration of Internet technology and family doctor-contracted services provides chronic disease patients in the community with more convenient and personalized health management services. After patients sign up with a family doctor team through an APP for their mobile phone, the family doctor can use the Internet platform to provide patients with online health consultation, health assessment, chronic disease follow-up, health guidance, and other services (23). At the same time, the family physician team can also understand the patient's health status in real time through the platform and adjust the management plan according to the patient's needs. Shenzhen's Shekangtong application is a prime example of this model in action. This platform serves as a central hub for all contracted residents. It not only facilitates secure video consultations and instant messaging with family doctors, but also integrates with smart devices (24). For a patient with hypertension, the platform automatically receives blood pressure readings from their home monitor, alerts the medical team, and provides personalized educational videos on managing sodium intake. Furthermore, it automates prescription refills and appointment scheduling. This deep integration allows for a truly refined treatment. For example, the Shenzhen model reported that among its contracted diabetic patients, the rate of achieving blood pressure control targets increased by 15%, and patient satisfaction scores for primary care services increased from an average of 75–92 100 within two years of the full implementation of the platform (25).

### 5.3 “Digitalization + self-management support” model

Digital technology provides a wealth of support tools and resources for self-management of chronic diseases. By developing a dedicated chronic disease self-management APP, patients can access disease knowledge, self-monitoring guidance, and healthy behavior interventions. The APP can also set reminders to help patients take their medications on time and monitor their condition regularly. At the same time, some APPs also provide social functions, where patients can exchange experiences and encourage each other on the platform to enhance their self-management confidence and motivation (26). A leading example is the “Tangyuan” diabetes self-management application, which has more than 2 million users in China. This app allows users to record their blood sugar, diet, and exercise information. Its key innovative feature is the use of AI for dietary analysis; users can take a photo of their meal, and the app will estimate the carbohydrate content and suggest portion sizes. The app generates personalized weekly health reports and posts popular science articles and expert lectures based on evidence on diabetes. It also incorporates gamification, awarding badges for consistent log-in and meeting step goals, and hosts a moderated community forum where users can share challenges and successes. A clinical study on the impact of the Tangyuan app, involving 5,000 users over six months, found that the average level of HbA1c (a key measure of long-term glucose control) decreased by 0.8%, and the self-reported ability of patients to manage their condition, measured by a standardized scale, improved by more than 30% (27).

## 6 Challenges and countermeasures of the digital empowerment community chronic disease management model

Digitalization can indeed change the traditional community chronic disease management model and has played a good role in practice. However, we must also note that digitalization is, after all, a new technology, and it will have two sides. We must pay attention to the challenges brought about by its widespread application.

### 6.1 Challenges faced by the chronic disease management model

#### 6.1.1 Data security and privacy protection issues

Digital management involves a large amount of patient personal health information, and data security and privacy protection are crucial. Once a data breach occurs, it will cause serious damage to the rights and interests of patients. Currently, there are still data security vulnerabilities and imperfect privacy protection measures in the data storage, transmission, and use process (28). For example, in 2022, a well-known health management app in China suffered a data breach that exposed the personal health information of millions of users, including their disease types and medication histories. This incident not only caused public panic but also significantly eroded the trust of patients in digital health platforms, highlighting the urgent need for robust security protocols.

#### 6.1.2 Imbalance in the application of digital technology

There are large differences in the construction of digital infrastructure and the talent pool of information technology between different regions and communities, leading to an imbalance in the application of digital technology in the management of chronic diseases in communities. In some economically underdeveloped areas and remote rural communities, digital equipment is scarce and medical personnel's information technology skills are low, making it difficult to effectively manage chronic digital diseases (29). A stark contrast can be seen between a community in Shanghai, where patients use advanced IoT devices for real-time health monitoring, and a rural community in western China, where community physicians still rely on paper records and manual follow-up due to the lack of network connectivity and digital devices, creating a significant “digital divide” in healthcare access.

#### 6.1.3 Patient acceptance and compliance issues

Some chronic disease patients, especially elderly patients, have limited acceptance of digital technology and have difficulty operating, leading to their low enthusiasm for using digital management tools and services. Furthermore, some patients have doubts about the effectiveness of digital management and have poor compliance, which affects the effectiveness of the implementation of digital management (30). A community health center in Beijing introduced a sophisticated chronic disease management app, but found that among patients over 65 years of age, the adoption rate was less than 20%. Many reported that the interface was too complex, the font was too small, and they were concerned about who could see their private health data, demonstrating a significant usability and trust barrier.

#### 6.1.4 Interconnection of information systems

Currently, the information systems between different medical institutions and between medical institutions and community health service centers have not yet fully achieved interconnection, and there are obstacles to data sharing. This makes the transmission of information untimely and inaccurate during the referral process, affecting the continuity and effectiveness of treatment (26). A common scenario is when a patient with hypertension is referred from a community clinic to a city hospital for specialist consultation. The hospital cannot access the patient's complete digital health records from the community, including long-term blood pressure trends and medication history, forcing the patient to undergo redundant tests and delay precise treatment due to these fragmented “data silos”.

### 6.2 Countermeasures for the chronic disease management model

In response to the challenges brought about by digital empowerment mentioned above in the management of chronic diseases in communities, the corresponding measures must be established.

#### 6.2.1 Strengthening data security and privacy protection

Establish and improve data security management systems and privacy protection regulations, and strengthen the supervision of data storage, transmission, and usage processes. Adopt advanced data encryption technology, access control technology, etc., to ensure the security and confidentiality of patient data. At the same time, strengthen data security and privacy protection training for medical personnel and relevant personnel to improve their awareness of safety and operational norms (31). For example, the provincial government of Zhejiang has piloted the use of blockchain technology on its regional health information platform. By storing patient data on a decentralized and immutable ledger, the platform ensures that all access to sensitive information is traceable and tamper-proof, significantly enhancing data security and patient trust.

#### 6.2.2 Promote the balanced development of digital technology

The government should increase investments in the construction of digital infrastructure in underdeveloped areas and remote rural communities, improve network communication conditions, and equip the necessary digital equipment. Conduct information technology talent training programs to improve the information technology skills of community medical staff. Encourage and guide technology companies to develop low-cost, easy-to-operate digital chronic disease management products and services suitable for use in grassroots communities and promote the widespread application of digital technology in the management of chronic diseases in communities (27). The “Digital Villages” initiative, part of China's national rural revitalization strategy, is a key countermeasure. It provides funding for villages to build 5G base stations and community digital health centers. Furthermore, tech companies are encouraged to develop simplified voice-activated health apps for elderly users, ensuring that the benefits of digitalization reach even the most remote areas.

#### 6.2.3 Improve patient acceptance and compliance

For groups such as elderly patients who have difficulty accepting digital technology, conduct special training and guidance, and help them become familiar with and master the use of digital management tools through face-to-face demonstrations and distribution of operation manuals. At the same time, optimize the interface design and operation process of digital management tools to make them more concise, easy to understand, and easy to use. Strengthen health education for patients, so that patients can understand the advantages and importance of digital management, and improve their trust and compliance with digital management (32). A community in Guangzhou has successfully addressed this by launching a “Digital Health Partner” program, where young volunteers provide one-on-one instruction to elderly residents on how to use health apps. They also helped co-design an app interface with larger fonts, intuitive icons, and voice-guided instructions, which increased the adoption rate among elderly patients by more than 50%.

#### 6.2.4 Promote the interconnection of information systems

Establish unified medical and health information standards and data interface specifications to promote interconnection and data sharing between the information systems of different medical institutions and community health service centers. Strengthen the construction of regional health information platforms to realize the centralized management and shared application of patient health information. Through the interconnection of information systems, improve the coordination and efficiency of medical services and provide patients with more convenient and continuous medical services (18). The city of Foshan in Guangdong province is an excellent example. It has established a unified “Foshan Health Information Platform” that connects all public hospitals, community health centers, and disease control centers within the city. When a patient is referred, his or her authorized health data, including medical history, allergies, and past test results, can be securely and instantly shared with the receiving physician, eliminating redundant procedures and enabling seamless and continuous care.

## 7 Conclusions

The innovation of the digital empowerment community chronic disease management model provides new paths and methods to deal with the increasingly serious challenges of chronic diseases. By building a digital health management platform, applying intelligent monitoring equipment, and carrying out mobile medical and telemedicine services, real-time monitoring, accurate assessment, and effective intervention of chronic disease patients have been realized, and the quality and efficiency of chronic disease management have been improved. The exploration and practice of innovative models such as AI + chronic disease management, Internet + family doctor contract service, and digital + self-management support have also achieved remarkable results, providing greater convenience and a better health management experience for patients with chronic disease. However, chronic disease management by the digital empowerment community still faces many challenges in the development process, such as data security, uneven application of technology, patient acceptance and compliance, and interconnection of information systems. Governments, medical institutions, technology companies, and all sectors of society need to work together and take effective measures to solve them. Looking toward the future, with the continuous development and integrated application of new generation information technologies such as 5G, artificial intelligence, big data, and the Internet of Things, the management of chronic diseases of the digital empowerment community will usher in a broader development space. We have reason to believe that with the help of digital technology, the community chronic disease management model will continue to innovate and improve, and play a more important role in protecting the health of residents and improving the national level of health.

Future research should move beyond technological implementation to address more nuanced and critical questions. First, there is a pressing need to conduct longitudinal studies on the long-term efficacy and cost-effectiveness of these digital models, evaluating their sustained impact on health outcomes and healthcare systems. Second, research must examine the ethical implications of AI in healthcare, particularly regarding algorithmic bias and its potential to exacerbate health disparities among different demographic groups. Finally, exploring human-centered design principles to improve digital literacy and accessibility for vulnerable populations, such as the elderly and those in remote areas, is crucial for ensuring equity. These directions will be vital for guiding the responsible and inclusive evolution of digital health management in China.

## Data Availability

The original contributions presented in the study are included in the article/Supplementary Material, further inquiries can be directed to the corresponding author.
